# Reducing psychological distress and depression in humanitarian emergencies: An essential role for nonspecialists

**DOI:** 10.1371/journal.pmed.1003625

**Published:** 2021-06-17

**Authors:** John A. Naslund, Eirini Karyotaki

**Affiliations:** 1 Department of Global Health and Social Medicine, Harvard Medical School, Boston, Massachusetts, United States of America; 2 Department of Clinical, Neuro- and Development Psychology, Section Clinical Psychology, Vrije Universiteit, Amsterdam, the Netherlands; 3 Amsterdam Public Health Research Institute, Faculty of Behavioural and Movement Sciences, Vrije Universiteit, Amsterdam, the Netherlands

## Abstract

John Naslund and Eirini Karyotaki discuss Mark Jordans and colleagues’ accompanying research study on therapy for people with psychological distress in Nepal.

Humanitarian emergencies such as war, natural disasters, or pandemics profoundly disrupt the daily lives of those impacted and result in psychological distress and high risk of mental disorders. With increasing frequency of humanitarian emergencies over the past decade, including the most recent Coronavirus Disease 2019 (COVID-19) pandemic, there is immediate need for brief scalable interventions that can be readily delivered to at-risk population groups [1]. With the dearth of available mental health specialists, especially in low-resource settings susceptible to crises, natural disasters, or displacement, combined with fragmented or poor functioning health systems during emergencies, nonspecialists may be ideally positioned to deliver such programs [2]. Nonspecialists, such as community health workers or lay persons, do not have specialty training in mental healthcare; yet, these frontline providers often play an essential role in delivering primary care services in many low- and middle-income countries [3,4], and they are increasingly being recognized as critical for scaling up access to psychological treatments for mental disorders [5,6]. Further, in a humanitarian crisis, use of nonspecialists from the affected population offers key benefits, such as empowering community members and drawing upon the experience of facilitators [7].

In an accompanying study in *PLOS Medicine*, Mark Jordans and colleagues demonstrate that community members with no prior mental health training could effectively deliver the WHO Group Problem Management Plus (Group PM+) program in a humanitarian setting in Nepal [8]. The research team conducted a cluster randomized controlled trial enrolling 72 wards and found that the 5-session Group PM+ delivered by nonspecialists contributed to reduction in psychological distress and depressive symptoms when compared to usual care. There may be opportunities to expand on these findings and further advance task sharing efforts in humanitarian settings.

## Skill use and the mechanism of action

Jordans and colleagues offer a novel exploration of why and how Group PM+ worked, revealing that participants’ use of the program skills such as breathing exercises, problem solving techniques, and seeking social support were important drivers in the difference in outcomes between study arms. This provides empirical evidence demonstrating which core components of the brief psychological intervention can achieve the desired outcome [8]. This adds to growing recognition of the need to unpack the multiple different elements that are combined into psychological treatment packages to yield greater specificity in determining what contributes to the target outcome [9]. Future studies will need to explore new avenues to optimize nonspecialist-delivered Group PM+ by varying the type and intensity of strategies aimed at promoting use of program skills. This could potentially be achieved through greater emphasis on practicing skills during the in-person group sessions, incorporating more opportunities for participants to receive feedback from program facilitators, and offering specific instruction for applying and tracking the ongoing use of these skills in day-to-day life for participants’ following program completion. Consideration of potential moderators is also important for determining whether there may be differential intervention response depending on individual characteristics, such as demographics, severity of distress, or history of mental health problems. Such insights could maximize treatment outcomes by targeting the use of Group PM+ for those who are more likely to benefit, while identifying at-risk individuals who may need additional services or professional help.

### Nonspecialist competency, supervision, and well-being

The authors ensured minimum level of competency of nonspecialists prior to being selected to deliver Group PM+ and employed a standardized supervision protocol for ongoing quality assurance. These important design strengths are essential to enable replication of quality intervention delivery and the positive study findings across other settings. However, nonspecialist supervision represents a major bottleneck to scaling up brief psychological interventions, given the costly requirement for expert supervisors, and continued reliance on in-person or group-based supervision [10]. While the assessment of nonspecialist competency using the ENACT scale is an important strength, this scale only covers general skills in delivering psychological interventions [11] and may not capture the treatment specific skills necessary to deliver Group PM+ effectively. Future avenues for supervising delivery of specific aspects of Group PM+ may also align with understanding of the mechanism of action. Nonspecialist supervision and assessment of competencies could reflect the importance of effectively teaching participants how to practice and apply the program skills in their day-to-day lives, as these appear essential for experiencing benefits.

Another often-overlooked aspect of nonspecialist delivered interventions is the mental health and well-being of the nonspecialists themselves. This is likely to be especially important in the context of humanitarian settings where the nonspecialists come from the same communities as the patients they serve and therefore have also experienced the same impacts of the disaster or emergency. There is mounting research showing that delivery of psychological treatments can contribute to risk of burnout, stress, and exhaustion [12,13], emphasizing the need for approaches to consider the needs of the nonspecialists. This may be critical for ensuring that they can continue to successfully deliver these programs in their communities.

### Role of technology for scaling up access

Technology may yield new opportunities to scale up access to programs when in-person contact is not possible, such as in conflict settings with significant security risks, or when the logistics are too difficult to coordinate, such as in rural areas or communities isolated due to natural disasters, and when in-person contact may not be permitted, such as during the COVID-19 pandemic [14]. Recent studies have reported on the increasing viability of digital approaches for supporting delivery of mental health services even in conflict settings and in severely resource-limited contexts at risk for humanitarian emergencies [15]; yet, there remains a substantial digital divide, particularly in impoverished communities and among women compared to men. Moreover, digital tools could allow opportunities to capture data from nonspecialists and participants to assess the treatment mechanism of action, facilitate remote supervision, and enable ongoing support for nonspecialists to ensure effective delivery of psychological interventions such as Group PM+. A key challenge will be adapting and translating nonspecialist-delivered psychological treatments to a digital format where clinical effectiveness is retained, while ensuring quality and safety for patients and equity of access given the challenging conditions in humanitarian crises.

## Conclusions

Jordans and colleagues’ study contributes compelling evidence on the effectiveness of a nonspecialist-delivered psychological intervention in a humanitarian setting, adding to prior research on the PM+ program in Pakistan [16] and Kenya [17], as well as studies of similar brief psychological interventions in refugees and asylum seekers in Uganda [18], Western Europe, and Turkey [19,20]. With nonspecialist-delivered psychological interventions showing promising outcomes across diverse settings, contexts, and cultures, our attention must now focus on sustaining quality delivery of these programs while scaling up access to reach the millions of people facing psychological distress and mental health consequences due to humanitarian emergencies globally. Continued efforts are needed to understand the mechanisms of action, recognize the needs of the nonspecialists themselves, and consider how technology can facilitate intervention delivery. Further consideration of the costs and cost–benefit of nonspecialist-delivered programs is also necessary to advocate for health systems and policymakers to prioritize access to these critically important services.
